# Cardiac magnetic resonance imaging of arrhythmogenic cardiomyopathy: evolving diagnostic perspectives

**DOI:** 10.1007/s00330-022-08958-2

**Published:** 2022-07-05

**Authors:** Alberto Cipriani, Giulia Mattesi, Riccardo Bariani, Annagrazia Cecere, Nicolò Martini, Laura De Michieli, Stefano Da Pozzo, Simone Corradin, Giorgio De Conti, Alessandro Zorzi, Raffaella Motta, Manuel De Lazzari, Barbara Bauce, Sabino Iliceto, Cristina Basso, Domenico Corrado, Martina Perazzolo Marra

**Affiliations:** 1 Department of Cardio-Thoraco-Vascular Sciences and Public Health, University of Padua, Via Giustiniani, 2, 35128 Padua, Italy; 2 Cardiology Unit, University Hospital of Padova, Padua, Italy; 3 Radiology Unit, University Hospital of Padova, Padua, Italy

**Keywords:** Arrhythmogenic right ventricular dysplasia, Magnetic resonance imaging, Sudden death

## Abstract

**Abstract:**

Arrhythmogenic cardiomyopathy (ACM) is a genetically determined heart muscle disease characterized by fibro-fatty myocardial replacement, clinically associated with malignant ventricular arrhythmias and sudden cardiac death. Originally described a disease with a prevalent right ventricular (RV) involvement, subsequently two other phenotypes have been recognized, such as the *left dominant* and the *biventricular* phenotypes, for which a recent International Expert consensus document provided upgrade diagnostic criteria (the 2020 “Padua Criteria”). In this novel workup for the diagnosis of the entire spectrum of phenotypic variants of ACM, including left ventricular (LV) variants, cardiac magnetic resonance (CMR) has emerged as the cardiac imaging technique of choice, due to its capability of detailed morpho-functional and tissue characterization evaluation of both RV and LV. In this review, the key role of CMR in the diagnosis of ACM is outlined, including the supplemental value for the characterization of the disease variants. An ACM-specific CMR study protocol, as well as strengths and weaknesses of each imaging technique, is also provided.

**Key Points:**

*• Arrhythmogenic cardiomyopathy includes three different phenotypes: dominant right, biventricular, and dominant left.*

*• In 2020, diagnostic criteria have been updated and cardiac magnetic resonance has emerged as the cardiac imaging technique of choice.*

*• This aim of this review is to provide an update of the current state of art regarding the use of CMR in ACM, with a particular focus on novel diagnostic criteria, CMR protocols, and prognostic significance of CMR findings in ACM.*

## Introduction

Arrhythmogenic cardiomyopathy is a genetically determined heart muscle disease characterized by fibro-fatty myocardial replacement, clinically associated with malignant ventricular arrhythmias (VAs) and sudden cardiac death [1].

Originally described in 1982 as *right ventricular dysplasia*, because of an abnormal development of right ventricular (RV) musculature, which was observed to be partially or completely substituted by fibro-fatty tissue [2, 3], subsequent studies focused on genetic and phenotypic characterization led to abandon the term *dysplasia* in favor of *cardiomyopathy*, in order to emphasize the crucial genetic contribute to the development of the disease [4]. In fact, genetic mutations in desmosomes, which are structures implicated in the intercellular junctions and adhesions, have been demonstrated to be involved in the pathogenesis of the disease [5], predisposing to myocyte detachment and apoptosis, and leading to progressive fibro-fatty myocardial replacement over time [6].

Although the first described phenotype was characterized by a massive RV involvement, well known as *arrhythmogenic right ventricular cardiomyopathy* (ARVC), in which RV morpho-functional abnormalities were predominant, two other phenotypes have been observed: the *left dominant variant* characterized by a predominant left ventricular (LV) involvement with no or minimal RV abnormalities, and the *biventricular variant* characterized by a parallel involvement of both ventricles [7, 8]. For these reasons, the designation *arrhythmogenic cardiomyopathy* (ACM) reflects better the concept of a genetic heart muscle disease, characterized by regional contraction abnormalities and fibrofatty myocardial replacement of one or both ventricles.

An accurate analysis of ventricular morphology and function is essential for the evaluation of patients and screening of relatives. Imaging techniques mostly used for ACM diagnosis include echocardiography and cardiac magnetic resonance (CMR). Echocardiography is the first imaging tool for the evaluation of patients with suspected ACM, but it has significant limitations in the assessment of RV and is not capable of myocardial tissue characterization. For these reasons, CMR has emerged as the imaging technique of choice in ACM, since it enables detailed morpho-functional and tissue characterization evaluation of both RV and LV.

Although the suspicion of ACM has become nowadays a frequent indication for CMR, which is very commonly prescribed in young individuals with cardiovascular symptoms, or electrocardiogram (ECG) abnormalities or premature ventricular beats, CMR laboratories familiar with ACM are limited and gaining experience with it can be hard due to the low prevalence of the disease. A basic understanding of the pathogenesis of ACM combined with the knowledge of technical principles of CMR is required for a reliable study execution and reporting. Incomplete protocols and incorrect interpretations of CMR images, leading to either false negative or false positive results, can have serious consequences for both patients and their families. With this review, we aimed to provide an update of the current state of art regarding the use of CMR in ACM, with a particular focus on novel diagnostic criteria, CMR protocols, and prognostic significance of CMR findings in ACM.

### The evolving diagnostic criteria for ACM and the increasing importance of CMR

The diagnosis of ACM is multiparametric, thus relying on multiple pathologic findings detected by different diagnostic modalities. Since 1994 [9], a diagnostic scoring system encompassing structural, histological, electrocardiographic, arrhythmic, and genetic factors is used to ascertain the diagnosis. Over the years, first in 2010 [10] and then in 2019 [11], the increasing clinical experience and knowledge of the disease led to critical appraisals and revisions of the criteria, with the intention to improve diagnostic accuracy.

Since the beginning, the role of CMR in the diagnosis of ACM has been recognized in the clinical demonstration of morpho-functional abnormalities caused by or reflecting the underlying histological changes of RV. In the first International Task Force (ITF) criteria of 1994, imaging criteria were mostly based on RV qualitative assessment and CMR was mentioned among the imaging modalities (alongside echocardiography and cardiac angiography) for the in vivo evaluation, despite the limited data and experience in this field [9]. The imaging criteria proposed showed poor reproducibility and low specificity, especially in familiar screening, because of the lack of any quantitative and objective standards for definition of abnormality [12]. Thus, the 2010 revised ITF criteria provided quantitative imaging reference values, based on sex specific volumetric measurements indexed to body surface area, to grade the severity of structural and functional RV abnormalities [10]. Moreover, the association of global RV dilatation or RV systolic dysfunction with regional wall motion abnormalities (i.e., akinesia or dyskinesia or dyssynchronous RV contraction) was deemed required in order to increase further the specificity.

After the inclusion of quantitative metrics in the structural criteria, CMR has gained importance in ACM evaluation, given its recognized high accuracy and reproducibility in chamber volume assessment [13] and great value in differential diagnosis with phenocopies [14]. Accordingly, the referral to CMR for suspected ACM has critically increased [15]. However, some limitations exist and should be kept in mind when dealing with ACM diagnosis. First, although important, CMR should never be used alone for ACM diagnosis, but always in combination with ECG, arrhythmic, and family data. This approach is helpful not only for exclusion of ACM mimics, but also for risk stratification. A previous work by te Riele et al demonstrated the utility of CMR to identify patients with ACM at high risk for arrhythmias, when used strategically in conjunction with ECG and Holter monitoring results [16]. CMR anomalies in the absence of ECG, arrhythmic, and genetic findings are inconclusive for ACM diagnosis and may be unhelpful for arrhythmic risk stratification. Second, variability in quantitative calculation of RV volumes in CMR, although lower than echocardiography or cardiac angiography, is inversely proportional to the experience of the reader, ranging from less than 10% in highly specialized centers to 20% in clinical practice [17]. So, great caution should be exercised in dealing with reports coming from not experienced CMR centers. Moreover, it should be noted that the reference values of normal RV end-diastolic volumes (up to 110 mL/m^2^ in men and 100 mL/m^2^ in women) were derived from 462 healthy volunteers of the multi-ethnic study of atherosclerosis, scanned with the traditional fast gradient echo cine sequences [10], which are known to underestimate volumes because of a lower contrast at the endocardial border for blood flow dependence and lower fat-to-myocardial contrast at the epicardial border. So, RV volumes calculated using the far more common today balanced steady-state free precession (bSSFP) images could be frequently higher than ITF reference values [18]. Also, the healthy volunteers used as controls had a mean age of 60 years, whereas it is well known that patients scanned for suspicion of ACM are instead represented by teenagers, most often athletes, presenting ventricular volumes well beyond the upper reference values established for RV, because of the physiologic adaptive remodeling typical of athlete’s heart [19, 20]. Third, although the most important feature of CMR is its capability of noninvasive myocardial tissue characterization, the detection of the pathologic hallmarks of ACM such as fibrosis or fat infiltration of RV, was not included among CMR diagnostic criteria, because of poor sensitivity and specificity, due to the thin wall of the RV and possible confusion with fat [21]. Fourth, LV phenotypes were not diagnosable with the 2010 ITF criteria, because of lack of evidences. So, the detection of fatty infiltration or non-ischemic late gadolinium enhancement (LGE) in LV, although common in ACM patients [22], was not useful for diagnosis.

All these limitations were reported in a critical review provided by an International Expert consensus document in 2020, in which the clinical performance of the available diagnostic criteria for ACM was investigated and potential areas of improvement were identified, like the absence of specific criteria for left-sided ACM variants and the lack of tissue characterization findings by CMR [11]. The recently published “Padua Criteria” [23] represent a further step toward the necessary upgrading of the criteria for diagnosis of the entire spectrum of phenotypic variants of ACM, which includes not only ARVC but also biventricular and left-dominant forms, and provides a modern clinical flow-chart for the diagnosis of ACM, for which CMR has assumed a key role [24].

### The 2020 ACM diagnostic criteria: the key role of CMR

The main novelty in the 2020 diagnostic criteria was the acknowledgement that pathogenic mutations, ECG abnormalities, or VAs are no longer sufficient for diagnosis of ACM, but at least one morpho-functional or structural RV or LV criterion, either major or minor, must be demonstrated for diagnosis of each phenotypic variant of ACM. This is a significant change of approach to the diagnosis of ACM, which now, like other structural heart muscle diseases such as hypertrophic and dilated cardiomyopathy, requires the demonstration of structural cardiac abnormalities, thus enhancing the role of cardiac imaging modalities. 

All modifications included in the 2020 diagnostic criteria concerning the use of CMR, and the rationale behind them are presented in Table 1.

**Table 1:** 2020 CMR diagnostic criteria for ACM and their rationale

Upgraded CMR diagnostic criteria	Rationale
A *major* morpho-functional criterion is fulfilled when regional wall motion abnormalities, such as RV akinesia, dyskinesia or bulging, are associated with either RV dilatation or dysfunction, regardless of their severity.	The distinction of morpho-functional criteria in major and minor, based on the severity of RV dilatation and dysfunction, is more useful for prognosis than for diagnosis.
RV regional wall motion abnormalities, such as akinesia, dyskinesia or bulging, in the absence of RV dilatation and/or systolic dysfunction, represent a *minor* morpho-functional criterion.	To increase in sensitivity in diagnosis of those ACM patients with RV wall motion abnormalities but normal size and systolic function. RV fibro-fatty lesions can be focal and limited in extension, thus altering the regional contractility without compromising the global RV size and function.
Demonstration of LV systolic dysfunction (by depression of LV ejection fraction or reduction of LV global longitudinal strain), with or without LV dilatation, is a *minor* morpho-functional criterion for diagnosing “biventricular” or “dominant-left” disease variants.	To diagnose LV involvement in ACM. Most patients show regional LV involvement without dilatation as a result of the segmental fibro-fatty scars.
Regional LV wall motion abnormalities, such as hypokinesia or akinesia (rarely dyskinesia) with a preserved LV systolic function is considered a *minor* morpho-functional criteria.	To diagnose LV involvement in ACM. Most patients show regional LV involvement without dilatation or systolic dysfunction as a result of the segmental fibro-fatty scars.
Cut-off values for RV and LV dilatation (normalized for sex and body surface area) and systolic dysfunction are those provided in the current nomograms of international societies of cardiovascular imaging [25]. In case of athletes, specific references values must be adopted [20]. See Table 3.	To promote a standardized image interpretation and post-processing in CMR laboratories. To acknowledge the physiologic adaptive changes of RV and LV in athlete's heart.
The detection of regional RV LGE is classified as a *major* structural myocardial criterion.	To increase specificity of RV wall motion abnormalities by demonstrating an underlying myocardial scar.
Demonstration of non-ischemic LV myocardial LGE/fibrosis is a *major* structural criterion for diagnosis of biventricular or left-dominant ACM.	To diagnose LV lesions in ACM.

*RV* right ventricle, *LV* left ventricle, *LGE* late gadolinium enhancement, *ACM* arrhythmogenic cardiomyopathy

These new criteria are heavily dependent on CMR, which has become necessary (although not sufficient) for diagnosis of ACM and characterization of disease phenotype, in particular those with LV involvement. In a recent CMR study, we characterized the ACM LV phenotype, pointing out its association with a large amount of non-ischemic LGE, affecting the subepicardial (less often the mid-myocardial) layers of the LV free wall, mostly the inferolateral region, with or without LV systolic dysfunction [22]. In some cases, subepicardial LGE can present a peculiar circumferential LV involvement (“ring pattern”), in particular when associated with genetic mutations in desmoplakin, desmin, and filamin-C [7, 26, 27]. Extent and distribution of LGE can help distinguish ACM LV phenotype from that of dilated cardiomyopathy [22], but not from that of other conditions like myocarditis, sarcoidosis, or neuromuscular dystrophies [28]. For this reason, the diagnosis of “left-dominant” ACM requires, in addition to consistent LV phenotypic features, the demonstration of a positive genotyping for ACM-causing gene mutation [23]. The genes having a definitive or moderate connection with right-dominant ACM (*PKP2*, *DSP*, *DSG2*, *DSC2*, *JUP*, *TMEM43*, *PLN*, *DES*) were recently identified in an international consensus [29].

To demonstrate how the 2020 Padua Criteria may offer the potential to change diagnostic end points and support the goal of improved health care, preliminary data of a comparative diagnostic analysis of a clinical ACM series using the 2010 vs. the new proposed criteria have been recently published [24]. These data showed that the clinical impact of the 2020 criteria was the increase of diagnostic sensitivity for ACM, and a better characterization of the disease phenotype, particularly those with LV involvement (biventricular and left dominant).

### CMR study protocol for ACM suspicion

Due to the multiple roles of CMR in diagnosis and prognosis of ACM patients, it is crucial to identify the more appropriate study protocol, personalized for each patient on the basis of family history, genotype, and clinical suspicion.

CMR study protocol for patients with suspected ACM should include cine sequences for LV and RV, black-blood, flow, and LGE images. The order of sequences and timing in relation to contrast administration can be modified according to laboratories preferences. Number of slices for technique must take in account the patient pre-test probability to have the disease, heart rate, rhythm, and capacity to maintain long and frequent breath-holds. Patients with two among ECG, echocardiography, arrhythmias or family criteria carry a high pre-test probability and deserve a longer and more accurate scan. In Table 2, protocol used in our institution as of today is presented.

**Table 2:** Study protocol for ACM patients in Padua CMR lab

CMR sequences		Imaging parameters	Mode of acquisition
Localizers			• Sagittal, coronal, transaxial
Cine CMR	Balanced steady-state free precession	Slice thickness 8 mm; TR/TE minimum, interslice gap 20%, flip angle 70°, parallel imaging 2.	• Long axis 4-ch, 3-ch, 2-ch view • Short axis stack (8–10 slices)
Cine CMR	Balanced steady-state free precession	Slice thickness 5 mm; TR/TE minimum, interslice gap 20%, flip angle 70°, parallel imaging 2.	• Right ventricular inflow (3–5 slices) • Sagittal RVOT (3–5 slices)
Black-blood CMR	T1- or proton density–weighted fast spin-echo	Slice thickness 8 mm; TR = 2RR; TE 25 ms parallel imaging 2.	• Long axis 4-ch, 3-ch, 2-ch view • Short axis stack (8–10 slices) • Right ventricular inflow* • Sagittal RVOT*
Edema CMR (optional**)	Turbo-inversion recovery magnitude	Slice thickness 8 mm; TR = 2RR; TE 76 ms; TI 160 ms parallel imaging 2.	• Long axis 4-ch, 3-ch, 2-ch view • Short axis stack (8–10 slices) • Right ventricular inflow * • Sagittal RVOT*
*GBCA administration*			
Cine CMR	Balanced steady-state free precession	Slice thickness 5 mm; TR/TE minimum, interslice gap 20%, flip angle 70°, parallel imaging 2.	• Right ventricular transaxial stack (8–10 slices)
Flow CMR (*optional; Recommended in case of RV dilatation*).	Through-plane motion-encoded phase-sensitive spoiled gradient echo	Slice thickness 6 mm; TR/TE 39/2.68; flip angle 20°	• Pulmonary artery • Aorta
Time inversion scout (about 8 min after GBCA administration)	Time-inversion scout gradient echo		• Mid short-axis view (or long-axis 4-ch view)
LGE CMR	Phase-sensitive inversion recovery gradient echo	TR/TE per manufacturer recommendations; slice thickness 8 mm; interslice gap 20%; Flip angle 25°; no parallel imaging. Use phase sensitive inversion recovery if available.	• Long axis 4-ch, 3-ch, 2-ch view • Short axis stack (8–10 slices) • Right ventricular inflow (3–5 slices) • Sagittal RVOT (3–5 slices)

*The study of RV should be more accurate the higher pre-test probability of RV disease is (echocardiography RV abnormalities, ECG V1-V3 Twave inversion, VA with LBBB morphology…)

**Useful in case of hot-phase presentation (chest pain/syncope/cardiac arrest plus troponin rise)

*CMR* cardiac magnetic resonance, *LGE* late gadolinium enhancement, *RV* right ventricle, *RVOT* right ventricular outflow tract, *GBCA* gadolinium-based contrast agent

#### Cine CMR

**Fig. 1:**
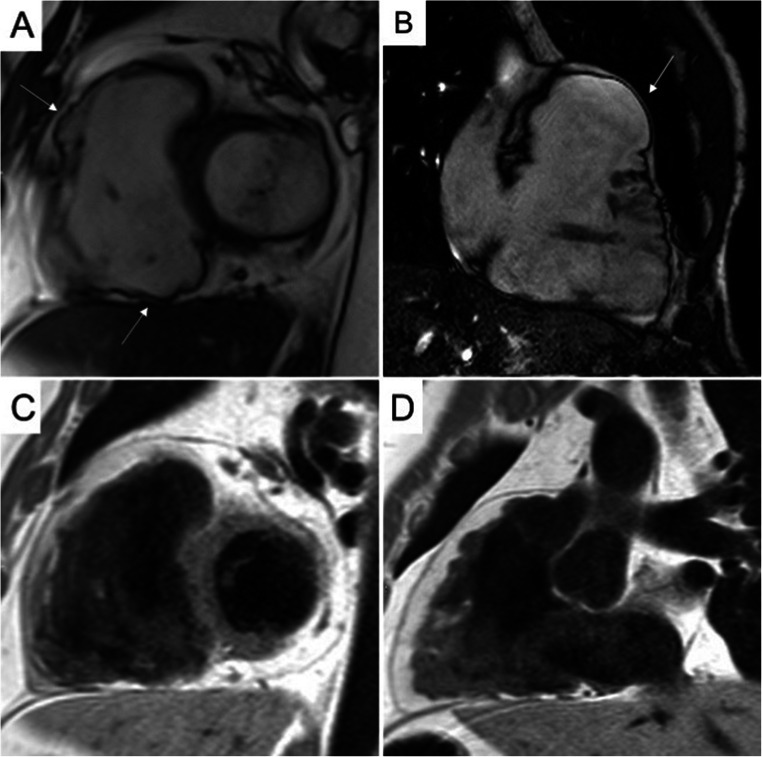
CMR images showing some peculiarities of a right dominant variant of ACM. **A**, **B** Cine image in short-axis view (systolic frame) showing multiple sacculations of the RV walls (white arrows). **C**, **D** T1-weighted images demonstrating massive fatty infiltration of the RV, with fat appearing as white tissue inside the RV wall. ACM, arrhythmogenic cardiomyopathy; RV, right ventricle

**Fig. 2:**
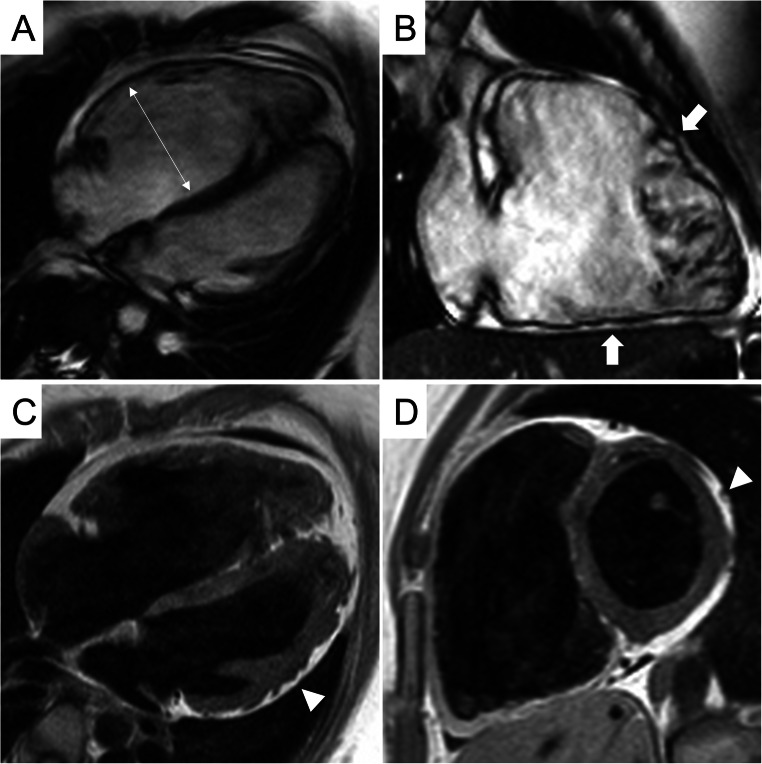
CMR frames of a patients with biventricular ACM. Morpho-functional abnormalities of the RV can be appreciated on 4-chamber view (**A**) and right ventricular 2-chamber long-axis view (**B**) of cine images, evidencing RV dilatation (**A**, double-head arrow) and multiple sacculations of the inferior and RVOT regions (**B**, arrows). Structural alterations of the LV are showed in 4-chamber view (**C**, arrowhead) and short-axis view (**D**, arrowhead) of T1-weigheted images, where fibro-fatty infiltration of the infero-lateral LV walls becomes evident as a hyperintense signal with a typical bite-like pattern. ACM, arrhythmogenic cardiomyopathy; LV, left ventricle; RV, right ventricle; RV, right ventricle outflow tract

The bSSFP sequences are characterized by a good contrast between endocardial borders and blood cavity, allowing a precise estimate of wall thickness, kinetics, biventricular volumes, and function. They have a central role in ACM evaluation, since they allow accurate wall motion assessment of RV and LV, in a reproducible and operator-independent manner. In Table 3, CMR cutoff values of EDV and EF for LV and RV of nonathletes and athletes, derived from the current nomograms of international societies of cardiovascular imaging, are provided. In addition to conventional cardiac imaging planes, like long- and short-axis, it is recommended to acquire also RV-dedicated images, like RV inflow, sagittal right ventricular outflow tract (RVOT), and transaxial, useful to detect focal RV akinesia, dyskinesia, and microaneurysms (Figs. 1 and 2). Some peculiar morphologic and kinetics anomalies have been described as typical of ACM, like the *accordion sign*, namely a contraction pattern of RV free wall, found more frequently in asymptomatic first-degree relatives of patients with ACM [30], and the *butterfly apex*, i.e., anatomic variation of the heart, which seems to have two separate apices [31]. Despite the peculiarity for ACM, caution should be used to define pathological these findings, especially when detected in isolation. Various normal anatomical variants exist, like pectus excavatum, which can be misinterpreted as findings of ACM leading to overdiagnosis. In doubtful cases, referral to experienced center for second opinion is highly recommended.

**Table 3:** Ventricular dilatation and systolic dysfunction by CMR in 2020 ACM Padua Criteria

Right ventricle dilatation and systolic dysfunction			
	Women	Men	Athletes
	IC 2020	IC 2020	IC 2020
EDV/BSA (mL/m^2^)	≥ 112	≥ 121	≥ 126
EF (%)	≤ 51	≤ 52	≤ 52
Left ventricle dilatation and systolic dysfunction			
	Women	Men	Athletes
	IC 2020	IC 2020	IC 2020
EDV/BSA (mL/m^2^)	≥ 96	≥ 105	≥ 119
EF (%)	≤ 57	≤ 57	≤ 57

Modified from Corrado et al [24]. All cutoff values refer to SSFP cine imaging technique. CMR cutoff values of EDV and EF for nonathletes (± 2 SD from the mean, respectively) are derived from Petersen et al [25] and for athletes (99% CI) from D’Ascenzi et al [20]

*CMR* cardiac magnetic resonance, *EDV* end-diastolic volume, *EF* ejection fraction, *BSA* body surface area

Feature-tracking CMR represents a promising tool for myocardial deformation analysis. In ACM patients, it may permit early diagnosis by detecting subtle wall motion abnormalities, in the absence of macroscopic dysfunction, especially in healthy gene carriers. Lastly, cine images can enable a first assessment of myocardial fat infiltration, in particular in LV variants [32].

#### Black-blood CMR

The T1- or proton density–weighted images are useful to identify fatty infiltration, visible as hyperintense signal in comparison to intermediate signal of normal myocardium. The research of myocardial fatty infiltration is motivated by the pathogenesis of the disease, which recognizes in the fibro-fatty lesion its pathologic hallmark [4, 8] (Fig. 1). However, the CMR detection of fat in the myocardium can be very challenging, due to a lower resolution of CMR images compared to endomyocardial biopsy or autoptic specimens [33]. In particular, fat imaging has a diagnostic sensitivity, specificity, and reproducibility lower in RV- than LV-ACM variants, because of the thinness of RV wall, and the common (normal) presence of adipose tissue in the outer layers of RV [20, 34]. In LV diseases, myocardial fat infiltration is more readily detected due to a greater wall thickness, and can show peculiar *bite-like* pattern, with regional overlap with LGE lesions [32] (Fig. 2).

Nevertheless, the presence of myocardial fat is *per se* neither a sign of disease nor a sign of ACM [35, 36]. Myocardial fat is not an uncommon finding on cardiac imaging in both healthy and diseased patients [37–39]. Physiological myocardial fat can be observed in the elderly and is typically located in the RV anterolateral and apical walls and outflow tract, in the absence of thinning of the RV myocardium [40]. In an autopsy study, RV myocardial fat was found in 85% of patients free of cardiac diseases [41]. Fatty infiltration can be also common in obese subjects with metabolic diseases and adipositas cordis [42], or can be detected in other pathologic conditions, like old myocardial infarction [43], dilated cardiomyopathy [44], neuromuscular dystrophies [45], lipomas, and tuberous sclerosis complex [46]. In patients with healed myocardial infarction, myocardial fat locates in a thinned left ventricular myocardium and follows the distribution of a coronary artery. The term “lipomatous metaplasia” defines the presence of myocardial fat within the myocardium which is not seen in the absence of substitutive myocardial fibrosis. Histological evidence of lipomatous metaplasia has been found in up to 68% of areas of left ventricular myocardial scars of patients who underwent transplantation for ischemic heart disease, but also in a substantial percentage of explanted hearts because of idiopathic dilated cardiomyopathy or chronic valvulopathy (24% and 37%, respectively) [47]. It generally correlates with extensive healed myocardial infarctions and is associated with severe heart failure. CMR imaging can identify this phenomenon by differentiating the various components of the necrotic myocardial tissue. Indeed, lipomatous metaplasia appears as a bright signal in T1-weighted spin-echo images that disappears with fat saturation, surrounded by the enhanced areas of myocardial fibrosis in post-contrast sequences [48]. T1 mapping technique is even more sensitive for lipomatous metaplasia in the context of ischemic scars, given its capability to accurately distinguish areas of lower native T1 values (fat) within areas with higher T1 (fibrosis). Although non-ischemic fibrofatty scars of ACM generally have lower extension and lower signal intensity than chronic myocardial infarctions, T1 mapping may be still useful in the distinction of the two components.

As a general rule, the evaluation of fat infiltration should be subordinated to morpho-functional assessment and scar analysis on cine and LGE CMR, respectively. The presence of myocardial fat should be used as a confirmation of diagnosis, rather than a criterion for diagnosis. Other techniques like the ECG-gated Dixon water-fat CMR are under investigation and appear promising in a more accurate visualization of fat infiltration [49].

#### Edema CMR

**Fig. 3:**
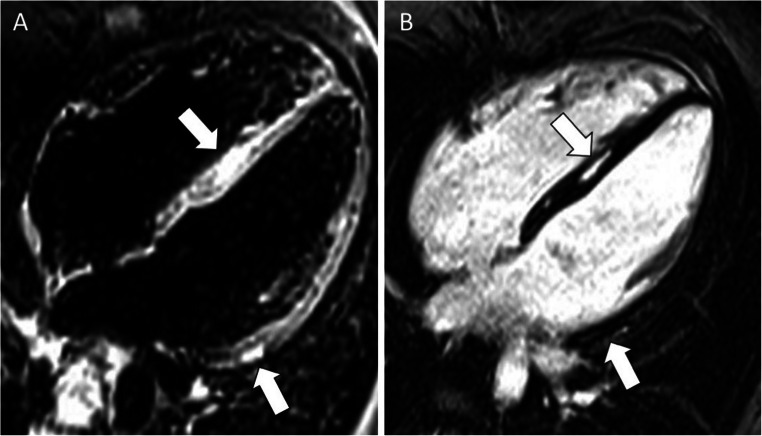
CMR images obtained from a patient during an episodes of “hot phase.” **A** The presence of edema (arrow) in the interventricular septum. **B** Inversion recovery sequences with late gadolinium enhancement (LGE) in the interventricular septum and lateral wall of left ventricle (arrows). Modified from Bariani et al [43]

The T2-weighted images are commonly used to depict myocardial edema in acute injuries of the myocardium. In patients with ACM, their routine use is not recommended, not for lower importance, but rather for shortening the scan time which would be too long, otherwise. They are, however, highly recommended in ACM patients presenting with *hot-phases*, an uncommon clinical presentation of ACM occurring more commonly in pediatric patients and carriers of desmoplakin gene mutations, characterized by chest pain and troponin release [50, 51]. In this context of myocarditis-mediated bouts of acute myocyte necrosis, inflammation and edema are characteristic features detectable by CMR. In a recent study by Bariani et al [50], 7 out of 12 patients (58%) presenting with acute phase showed myocardial edema (Fig. 3). In addition, T2-weighted with fat suppression images may serve as confirmation of fat infiltration, in those regions presenting a hypointense signal, evidenced as hyperintense in T1-weighted images.

T2 mapping is another technique useful for the study of acute myocardial injuries, given a high sensitivity for edema and inflammation. Chun et al recently investigated the application of T2 mapping in ACM patients, and demonstrated an association of high T2 values and heart-failure events (a composite of hospitalization, heart transplantation, and cardiac death due to systolic dysfunction) during follow-up [52].

#### Flow CMR

**Fig. 4:**
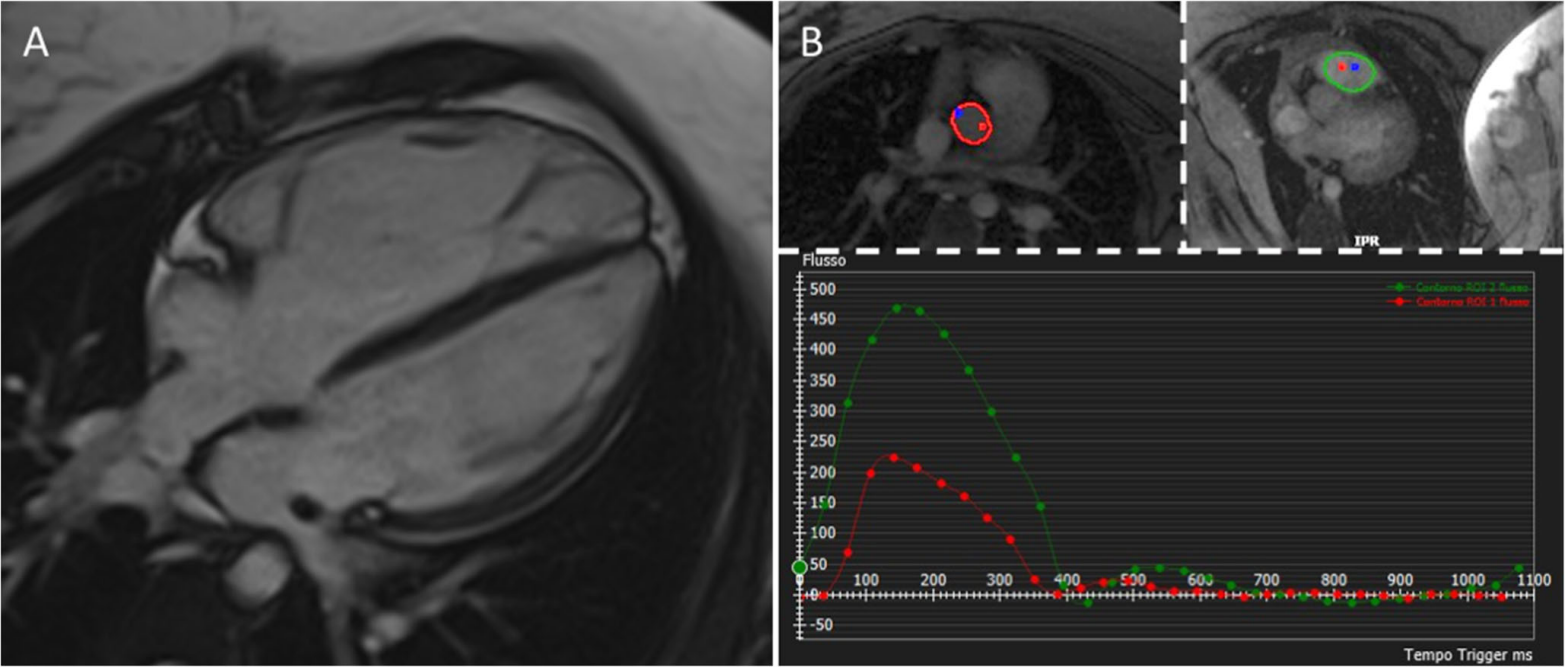
CMR images in a 21-year-old admitted for effort-related syncope. **A** A severe RV dilatation. **B** Flow CMR demonstrated the differences in cardiac output between aorta and pulmonary artery, suggestive of left-to-right shunt (Qp/Qs 2.5). The patient was diagnosed with atrial septal defect (upper sinus venosus type) and partial anomalous pulmonary venous drainage

Through-plane motion-encoded phase-sensitive spoiled gradient echo is a useful CMR technique to assess blood flow. In cardiology, they have an indication in valve regurgitation assessment, ventricular output, cardiac shunt, and Qp/Qs estimate. Flow CMR is optional in patients with ACM; however, in the presence of RV dilatation, it is highly recommended to exclude congenital heart diseases, having left-to-right shunt causing RV volume overload, like interatrial septal defects, anomalous pulmonary venous drainages (Fig. 4), or patent ductus arteriosus.

#### LGE CMR

Eight to 10 min after the gadolinium-based contrast agent administration, phase-sensitive inversion recovery gradient echo sequences permit the visualization, if present, of LGE, such as necrotic or fibrotic myocardium areas which assume a bright signal (hyperenhancement), compared to the remote (healthy) myocardium, which remains dark. The pattern of LGE is useful to differentiate post-infarction necrosis (subendocardial or transmural LGE) from fibrosis in non-ischemic cardiomyopathies (mid-wall LGE, subepicardial LGE), or myocarditis (mid-myocardial, subepicardial, or focal LGE).

**Fig. 5:**
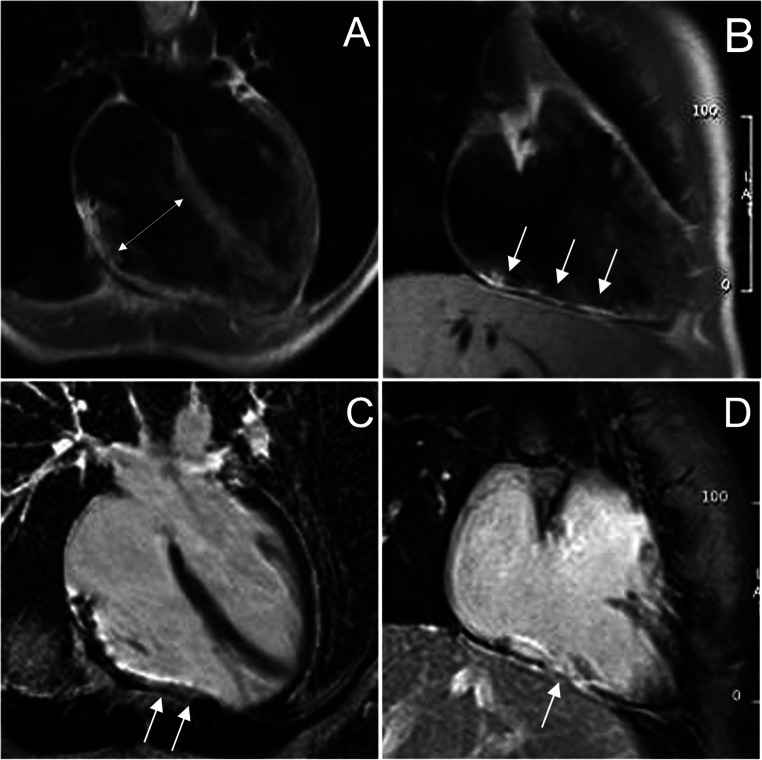
CMR images in a patient with a right dominant ACM. T1-weighted (**A**, **B**) and post-contrast images (**C**, **D**) showing the dilatation (**A**, arrow) and the fibro-fatty infiltration (arrows, **B**, **C**, **D**) of the free and diaphragmatic wall of the RV. ACM, arrhythmogenic cardiomyopathy; RV, right ventricle

**Fig. 6:**
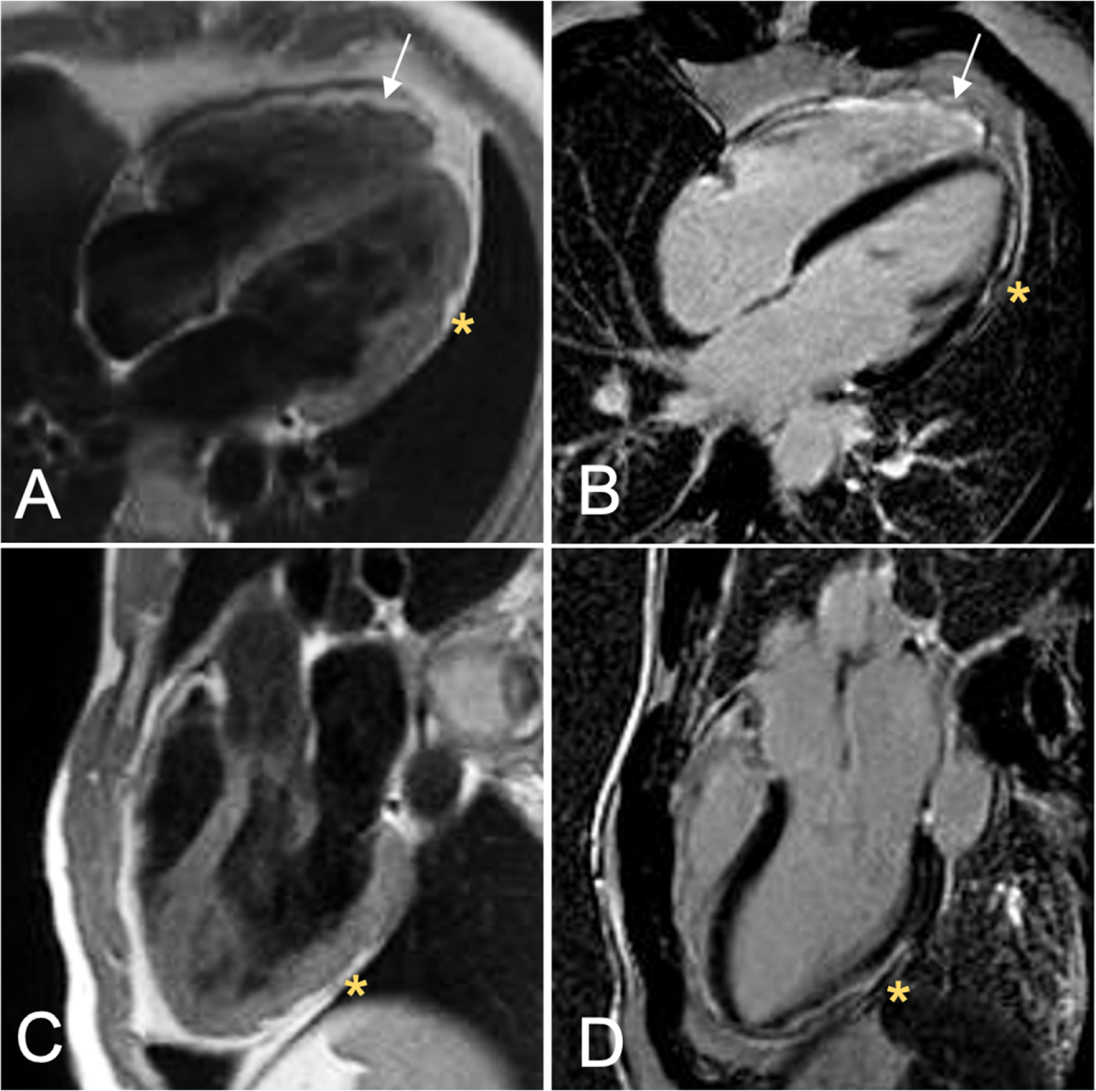
CMR in a patient with biventricular ACM demonstrating the fibro-fatty infiltration in the RV free wall (**A**, **B**; arrow) and the concomitant involvement of the LV lateral wall (**A**–**D**, asterisks). In details, T1-weighted (**A**) and post-contrast images (**B**) four chamber views showed the fibro-fatty infiltration of the LV antero-lateral wall (**A**, **B**; yellow asterisks) and of the apical RV wall (**A**, **B**; white arrows) and T1-weigheted (**C**) and post-contrast sequences (**D**) three chamber views showed that also the LV infero-lateral wall (**C**, **D**; yellow asterisks) was involved by the disease. ACM, arrhythmogenic cardiomyopathy; LV, left ventricle; RV, right ventricle

In ACM patients, LGE technique permits the visualization of the hallmark lesions of ACM, which consist of ventricular fibro(-fatty) myocardial replacement. Furthermore, it can help categorize the ACM phenotypic variant [23], and risk stratify patients for arrhythmic events, particularly those with left-dominant forms [53]. However, *all that glitters is not gold* and LGE assessment in ACM needs some caveat. Literature data indicate that in RV diseases, LGE has a variable sensitivity, being appreciable in about 30–70% of patients, with possible mismatch when compared to electroanatomic-voltage mapping or endomyocardial biopsy findings [7, 54–57] (Fig. 5). This limited yield of positive RV LGE may be due to the low resolution of the current LGE technique for the RV wall, which has a limited thickness and is surrounded from hyperintense signals coming from blood cavity and adipose tissue, but can be improved when considering LGE together with wall motion abnormalities [58]. By contrast, in RV diseases, LGE is identifiable in LV of half to two-thirds of patients, commonly affecting the subepicardial layers of the LV free wall, mostly the inferolateral region, with or without septal involvement [22, 32] (Fig. 6). Importantly, the presence of LV LGE may not associate with wall motion abnormalities, being mostly confined to the outer wall layers, which are those contributing less to the systolic contraction. As a corollary, LV disease cannot be diagnosed by imaging modalities focused only on wall motion assessment and LV function.

Since many other cardiac conditions can present non-ischemic LGE patterns, like dilated cardiomyopathy, myocarditis, neuromuscular dystrophies, and sarcoidosis, caution should be used to conclude as diagnostic for ACM any LGE finding [28]. Again, in doubtful cases, referral to experienced center for second opinion is recommended, taking in mind that CMR data alone are not conclusive for ACM diagnosis.

## Native T1 mapping and extra-cellular volume

Native T1 mapping and extra-cellular volume (ECV) quantification are useful imaging techniques able to identify an increase of interstitial space, due to diffuse fibrosis or infiltration, particularly helpful in the differential diagnosis of cardiomyopathies with hypertrophic phenotype [59]. Although myocardial fibrofatty lesions in ACM are more commonly described as focal abnormalities, the diagnostic yield of native T1 mapping has been recently investigated also in these patients [60]. Bourfiss et al studied native T1 mapping and its dispersion in genotype-positive ACM patients, relatives, and controls and found that genotype-positive ACM patients had significantly higher native T1 values than controls, suggesting a predominant role of LV replacement fibrosis rather than fat infiltration (that usually reduces T1 values) in ACM pathogenesis. Conversely, both genotype-positive ACM patients and at-risk relatives showed a greater T1 dispersion than controls, probably due to regional microstructure changes, more commonly located in the posterolateral and inferior regions [61].

It has been also proposed that T1 changes may precede LV focal abnormalities in ACM and favor early diagnosis. Georgiopoulos et al indeed demonstrated an abnormal increase of T1 values in 11/30 ACM patients (37%), including those with normal LV at conventional imaging tests [62].

### Prognostic role of CMR in ACM

Besides the diagnostic purposes, CMR findings can also help risk stratify ACM patients for arrhythmic events and guide life-saving therapy as implantable cardioverter-defibrillator (ICD) implantation. In the 2015 International Tack Force paper, severe RV dilatation and dysfunction are considered among the strongest predictors of malignant arrhythmias and therefore primary prevention ICD implantation is recommended in these patients regardless of arrhythmic burden [63]. Furthermore, it has been demonstrated that the presence of any abnormalities at CMR such as RV and/or LV fat infiltration identifies patients with increased risk of arrhythmic events and worse outcome [64]. The prognostic role of LGE in ACM patients is well defined since 2005. Tandri et al evaluated 30 compared findings of contrast-enhanced CMR with those resulting from electrophysiological study and endomyocardial biopsy. The authors concluded that the presence of RV LGE correlated with the inducibility of arrhythmias on electrophysiological study [55]. More recently, the role of CMR was evaluated within a multi-parameter scoring approach by Aquaro et al [53]. In this study, an evaluation of wall motion abnormalities, chamber sizes, function, and tissue characterization in both ventricles was performed. Based on phenotypic expression, the cohort of patients was then classified in patients with isolated RV disease, isolated LV disease, biventricular disease, and no structural disease. Authors concluded that different CMR phenotype of ACM are associated with different prognoses, underlying that patients with LV LGE had a worse prognosis than those with lone RV disease [53]. Finally, as well as for T2, also high values of native T1 and ECV recently emerged as predictors of adverse outcome during follow-up [52].

## Conclusion

The traditional classification of cardiomyopathies relied on traditional diagnostic techniques like echocardiography and angiography, able to identify morpho-functional anomalies of the heart like hypertrophy, dilatation, and restriction. However, in the last decades, it is emerging that information coming from CMR myocardial tissue characterization (i.e., inflammation, fibrosis, fibro-adiposis…) are crucial for a better understanding of cardiomyopathies and for improvement of diagnostic accuracy and risk stratification. In particular, CMR shed light upon ACM, because it allowed to characterize the disease phenotype, particularly the biventricular and left-dominant ones, to exclude phenocopies and risk stratify patients. The Padua Criteria underscore the increasing clinical experience with the expanding spectrum of ACM phenotypes and aim to improve the diagnosis of ACM, particularly by means of CMR. Like previous ITF criteria, also the Padua Criteria need to be further validated by clinical studies in large cohorts of “real word” patients.

## Abbreviations

ACM: Arrhythmogenic cardiomyopathy
ARVC: Arrhythmogenic right ventricular cardiomyopathy
bSSFP: Balanced steady-state free precession
CMR: Cardiac magnetic resonance
ECG: Electrocardiogram
ITF: International Task Force
LGE: Late gadolinium enhancement
LV: Left ventricle
RV: Right ventricle
RVOT: Right ventricular outflow tract
VA: Ventricular arrhythmias

## Declarations

### Guarantor

The scientific guarantor of this publication is Prof. Domenico Corrado.

### Statistics and biometry

No complex statistical methods were necessary for this paper.

### Methodology

• not applicable

• not applicable

• performed at one institution
